# Dermal Delivery of Niacinamide—In Vivo Studies

**DOI:** 10.3390/pharmaceutics13050726

**Published:** 2021-05-14

**Authors:** Yanling Zhang, Chin-Ping Kung, Fotis Iliopoulos, Bruno C. Sil, Jonathan Hadgraft, Majella E. Lane

**Affiliations:** 1 Department of Pharmaceutics, University College London School of Pharmacy, 29-39 Brunswick Square, London WC1N 1AX, UK; c.kung@ucl.ac.uk (C.-P.K.); fotis.iliopoulos.16@ucl.ac.uk (F.I.); j.hadgraft@ucl.ac.uk (J.H.); m.lane@ucl.ac.uk (M.E.L.); 2 School of Human Sciences, London Metropolitan University, 166-220 Holloway Road, London N7 8DB, UK; b.dasilvasildossantos@londonmet.ac.uk

**Keywords:** niacinamide, skin permeation, in vivo, confocal Raman spectroscopy, tape stripping, in vitro-in vivo correlation

## Abstract

In vivo human studies are considered to be the “gold standard” when investigating (trans)dermal delivery of actives. Previously, we reported the effects of a range of vehicles on the delivery of niacinamide (NIA) using conventional Franz cell studies. In the present work, dermal delivery of NIA was investigated in vivo in human subjects using confocal Raman spectroscopy (CRS) and tape stripping (TS). The vehicles investigated included propylene glycol (PG), Transcutol^®^ P (TC), binary combinations of PG with oleic acid (OA) or linolenic acid (LA) and a ternary system comprising of TC, caprylic/capric triglyceride (CCT) and dimethyl isosorbide (DMI). For the CRS studies, higher area under curve (AUC) values for NIA were observed for the PG:LA binary system compared with PG, TC and TC:CCT:DMI (*p* < 0.05). A very good correlation was found between the in vitro cumulative permeation of NIA and the AUC values from Raman intensity depth profiles, with a Pearson correlation coefficient (R^2^) of 0.84. In addition, an excellent correlation (R^2^ = 0.97) was evident for the signal of the solvent PG and the active. CRS was also shown to discriminate between NIA in solution versus crystalline NIA. The findings confirm that CRS is emerging as a powerful approach for dermatopharmacokinetic studies of both actives and excipients in human.

## 1. Introduction

To establish the efficacy and safety of dermal drug delivery systems, assessment of the skin penetration of actives and, increasingly, formulation components is of critical importance [1,2]. In vivo human studies are the most relevant and preferred approaches to understand percutaneous delivery of actives in human. The early in vivo studies on percutaneous penetration in human subjects were conducted with radiolabeled actives because of the limitations in analytical methodology for non-labeled compounds [3,4]. Tape stripping (TS) has also been proposed as a method to study in vivo percutaneous absorption and involves application of formulations to the skin, followed by sequential removal of the outermost skin layer, the stratum corneum (SC), by adhesive tapes [5]. In addition to determining uptake of topically applied actives, TS has also been used for measurement of skin barrier function [5]. To measure percutaneous absorption using TS, the amount of active substance retained in the tapes after stripping the SC must be extracted and quantified [6]. These additional procedures for sample extraction and analysis inevitably mean that the TS technique is time consuming and laborious. In 1998, the United States Food & Drug Administration (FDA) published a draft guideline on TS for assessment of the bioavailability/bioequivalence of topical formulations [7]. However, the guideline was withdrawn a few years later because of contradictory results from different laboratories [8]. Variability in results from different TS studies has also been ascribed to differences in the amounts of SC removed for the specific brand of tape used [9]. In addition, TS is operator-dependent as the pressure applied during application and the force exerted in removal of tapes introduces further variation to the sampling procedure [10]. Finally, given that only superficial layers of SC are removed during the procedure, TS is limited to measurements of the local amounts of drug in the SC itself [11]. Therefore, alternatives to TS for evaluation of topical delivery of actives and evaluation of bioequivalence of topical formulations in human are desirable.

Spectroscopic methods offer a number of advantages for assessment of drug delivery to the skin [12]. Confocal Raman spectroscopy (CRS), with reference to in vivo measurements, combines the principles of confocal microscopy with Raman spectroscopy. The application of this method for analysis of skin at the molecular level was first reported by Caspers and coworkers [13,14]. In these studies, the authors investigated the use of CRS for non-invasive depth profiling of SC water content and other endogenous components, including natural moisturizing factor. Boncheva et al. [15] evaluated the hydration profile of the SC using conductance measurements combined with TS, compared with in vivo CRS. CRS was confirmed as a more suitable approach for such studies because of the non-invasive nature of the technique and lack of variability compared with conductance measurements. The ability of CRS to advance our understanding of in vivo skin delivery of various active substances and solvents was later reported by a number of researchers [16,17,18,19,20]. Pudney and coworkers reported that the skin penetration of the active *trans*-retinol in human volunteers was highly correlated with the depth of penetration of the vehicle for the compound, namely propylene glycol (PG). Mohammed et al. [17] reported the first study that demonstrated a correlation between in vivo CRS studies and conventional in vitro skin permeation data for the model active niacinamide (NIA). Results for NIA permeation in human epidermis mounted in diffusion cells were compared with CRS data for skin uptake of the compound. An excellent correlation (r^2^ = 0.96) was reported for the cumulative permeation of NIA in vitro and the signal intensity of NIA detected in the SC in vivo using CRS. However, the results from this proof-of-concept study were obtained in one single subject and further studies with a lager sample size are clearly needed to probe the in vitro-in vivo correlations observed.

NIA is the water-soluble form of vitamin B3 and has been widely used in topical formulations because of its proven efficacy in the management of various skin disorders, including hyperpigmentation [21] and inflammation [22], as well as the ability of NIA to increase skin barrier function [23] and prevent UV-induced immunosuppression [24]. Recently, we conducted further in vitro permeation studies to identify suitable candidate vehicles for dermal delivery of NIA [25,26]. The highest in vitro permeation of NIA was observed for two binary solvent systems, PG/oleic acid (OA) and PG/linolenic acid (LA), respectively. To understand further the effects of such formulations on the enhancement of NIA skin penetration in human, in vivo human experiments are desirable. To this end, both TS and CRS were performed to study the permeation of NIA and PG into SC involving six healthy volunteers. Another aim of this study was to compare the in vivo data obtained by TS and CRS approaches with previously published in vitro data from Franz cell diffusion studies in human skin. PG:OA (10:90), PG:LA (50:50), Transcutol^TM^ (TC):caprylic/capric triglyceride (CCT):dimethyl isosorbide (DMI) (50:25:25) and two neat solvents, namely TC and PG. These vehicles were taken forward based on pre-formulation data as well as screening of the solvents in a range of skin models [25,26]. Additionally, these materials are generally recognized as safe (GRAS) and widely used in pharmaceutical and personal care products [27,28,29].

## 2. Materials and Methods

### 2.1. Materials

NIA, OA and LA were purchased from Sigma Aldrich, Dorset, UK. High performance liquid chromatography (HPLC) grade water, methanol and PG were obtained from Fisher Scientific, Leicestershire, UK. DMI was supplied by Croda Ltd., Goole, UK. TC and CCT were generous gifts from Gattefossé, Saint-Priest, France. Standard D-Squame tapes (diameter: 2.2 cm; area: 3.8 cm^2^) were purchased from CuDerm Corporation (Dallas, TX, USA).

### 2.2. Study Design

The study was conducted with approval from the Research Ethics Committee, University College London (Reference number REC 13271/001) on 14 December 2018. Following written informed consent, six volunteers (age 27.2 ± 1.7 years; 4 males and 2 females) were enrolled in the study. Five vehicles containing 5% (*w/v*) of NIA were evaluated in this in vivo project: two neat solvents PG and TC; two binary systems PG:OA (10:90) and PG:LA (50:50); and one ternary system TC:CCT:DMI (50:25:25). Three well-separated investigation sites (3.8 cm^2^) were delineated on the volunteers’ volar forearms, for a total of five application sites and one control site. An amount of 10 μL (2.6 μL/cm^2^) of the respective NIA vehicle was applied on each site using a micropipette. CRS measurements and tape stripping were conducted 1 h after the application. The skin surface was gently cleaned using a cotton bud before any measurement. There was a minimum of a 14-day wash out period between CRS and TS studies.

### 2.3. Confocal Raman Spectroscopy (CRS) Studies

Raman measurements were performed in vivo using a Model SCA 3510 confocal Raman spectroscopy skin composition analyzer (RiverD International B.V., Rotterdam, The Netherlands) as reported previously [17,30]. Two lasers, 785 and 690 nm, were coupled to the CRS instrument via fiber optic cables. These wavelengths allowed measurements in the fingerprint region (400–1800 cm^−1^) and high wavenumber region (2500–4000 cm^−1^) of the Raman spectrum, respectively. On the day of the experiment, the instrument was calibrated using a National Institute Standards glass standard following the procedure described by Iliopoulos et al. [30], prior to any measurements. The water content in the SC was determined by analyzing the high wavenumber Raman spectral data within 2600–3800 cm^−1^ (SkinTools V 2.0 R120802, RiverD International, B.V., Rotterdam, The Netherlands). Water concentration profiles were measured based on the ratio of the Raman signals from the protein and water molecules, followed by the integration of the corresponding signals of the obtained spectra at measured depth increments [14,31]. Subsequently, SC thickness was estimated based on the water concentration profiles as described in the literature [32].

Raman spectra in the fingerprint region were measured using the 785 nm laser. A 5 s exposure time was used with 2 μm steps to a final depth of 30 μm. To minimize the biological lateral variation in skin composition, three frames were taken at each measurement position and the spectra were averaged. Data acquisition was conducted using RiverICon V 3.0.130327 software (RiverD International B.V., Rotterdam, the Netherlands). The reference spectrum of NIA required for the fitting algorithm was obtained with a 5% NIA aqueous solution and the water signal was subsequently subtracted. The Raman spectra collected from the investigation sites were fitted based on the endogenous SC components with reference spectra [31]. The depth of the measurements was normalized as described elsewhere [18]. The thickness of the SC measured (x) was divided by the total SC thickness (h) for the value of the normalized SC thickness to fall in the range of 0 ≤ x/h ≤ 1. This allowed the depth profiles of the active/excipient to be expressed as a function of the relative position within the SC (x/h), removing inter-subject variability. The area under curve (AUC) was calculated for NIA profiles using trapezoidal integration.

### 2.4. Tape Stripping (TS) and Transepidermal Water Loss (TEWL) Measurement

The NIA distribution across the SC following application of the formulations was investigated by sequential removal of the outer skin layers using TS. Standard D-Squame tape^®^ was used in this study, and 15 successive tapes were collected from each site. A constant pressure was applied on each tape using a pressure device for 5 s (225 g/cm^2^) as described previously [24,33,34]. The first tape was discarded and the remaining tapes (Tapes 2–15) were collected in 2 mL Eppendorf^®^ tubes with 1 mL of methanol and extracted overnight and the amounts of NIA were subsequently quantified [26]. The TEWL (Aquaflux AF102, Biox System Ltd., London, UK) measurements were made at the control site at baseline and after the removal of the first, third, sixth, ninth, twelfth and fifteenth tape [35].

### 2.5. Data Analysis

All results are reported as the mean ± standard deviation (SD). Statistical analysis was performed using SPSS^®^ Statistics Version 24 (IBM, Feltham, UK). The Shapiro–Wilk Test was adopted to examine the normality of data and Levene’s Test was used to assess the homogeneity of variance. One-way ANOVA was performed for data that met the assumptions of normality and homogeneity of variance. Tukey’s HSD post hoc test was used post ANOVA analysis. The Kruskal–Wallis H Test was used for non-parametric data or where the assumption of homogeneity of variance between groups was violated in the ANOVA analysis. A *p*-value lower than 0.05 (*p* < 0.05) was considered as a statistically significant difference. Correlations were determined using the Pearson product correlation coefficient (R^2^) using MS Excel (Microsoft Corp., St. Redmond, WA, USA).

## 3. Results and Discussion

### 3.1. CRS Studies

**Figure 1:**
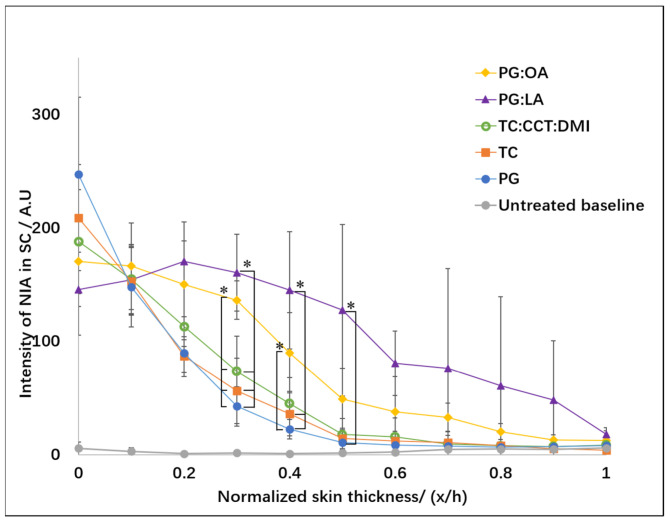
NIA signal intensity depth profiles in the SC 1 h after application of five vehicles, namely PG, TC, PG:OA, PG:LA and TC:CCT:DMI (mean ± SD, *n* = 6, * *p* < 0.05).

Six healthy volunteers were recruited for this study. The mean SC thickness of their volar forearms was determined as 18.4 ± 4.2 μm. These values are in line with values reported in the literature [33,34]. The depth profiles for NIA signal intensity in the SC 1 h after application of formulations are shown in Figure 1.

Significantly higher signal intensity values of NIA were detected at the skin surface (x/h = 0) compared with the deeper SC layers (0.6 ≤ x/h ≤ 1) for PG, TC and TC:CCT:DMI (*p* < 0.05). Differences were also evident for the NIA signal intensity at x/h = 0.1 compared with the intensity at 0.8 ≤ x/h ≤ 1 for PG, TC and TC:CCT:DMI (*p* < 0.05). As shown in Figure 1, similar profiles were noted for PG and TC, where the NIA signal decreased exponentially as a function of depth, as is typical for non-steady state diffusion. The profile for TC:CCT:DMI appears to be linear between the skin surface (x/h = 0) to a depth of 0.5 (x/h). For the binary PG:OA systems, a higher NIA signal was evident in the upper tissue layers (x/h = 0 and 0.1) compared with the NIA signal for PG:OA in the lower SC layers (0.8 ≤ x/h ≤ 1, *p* < 0.05). Comparing the NIA Raman signal intensity values for all formulations, differences were only noted at depths of 0.3 and 0.4 (x/h) for the PG:LA and PG:OA systems. Overall, higher values of NIA intensity were evident for PG:LA and PG:OA compared with the other vehicles (x/h = 0.3, *p* < 0.05). At a depth of 0.4 (x/h), greater NIA signal intensities were determined for PG:LA compared with TC and PG (*p* < 0.05), as well as for PG:OA compared with neat PG (*p* < 0.05).

**Figure 2:**
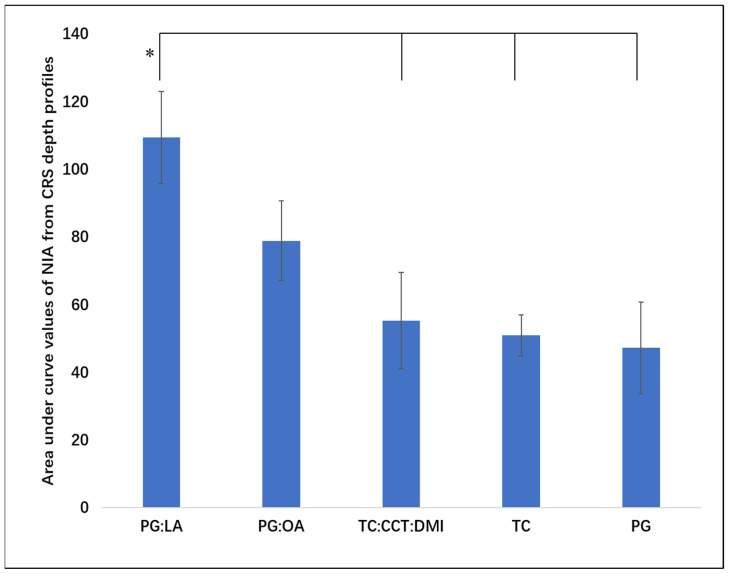
Average area under curve (AUC) values for NIA CRS intensity depth profiles (mean ± SD, *n* = 6, * *p* < 0.05).

The corresponding AUC values for NIA CRS intensity depth profiles are shown in Figure 2. Higher values of the AUC were confirmed for the PG:LA binary system compared with PG, TC and TC:CCT:DMI (*p* < 0.05). No significant difference was observed for the AUC values of NIA for PG:LA and PG:OA (*p* > 0.05). These results are in line with the synergistic enhancement noted for binary systems composed of PG and fatty acids previously reported in vitro in human skin. [26]. In this 24 h finite dose study, PG:OA and PG:LA promoted significant enhancement of NIA penetration compared with neat PG.

The mechanisms of action for PG and fatty acids were systematically reviewed by Lane [35]. PG is a solvent that is widely used in topical formulations, and the uptake of PG in human skin has been reported previously [36,37,38]. The solubility of NIA in PG has been previously reported as 28% (*w/v*) at 32 °C [25]. The skin uptake of PG may modify the solubility properties of the skin and therefore increase NIA permeation [36,39]. Fatty acids such as OA and LA may modify skin permeation by affecting the diffusion of permeants. OA has one double bond in the cis configuration, forming a “kinked” shape. Additionally, LA has three cis double bonds that limit the conformational freedom of the alkyl chain. These conformations have been suggested to promote disruption of SC lipid packing by introducing a separate phase in the intercellular lipid domain, that may contribute to increased diffusion of permeants [40]. Although skin permeation enhancement by combining PG with fatty acids has been noted in several studies for different compounds [26,41,42], the exact synergistic mechanism of PG and fatty acids is not fully understood. It might be hypothesized that permeable defects created by fatty acids may increase the diffusion of PG with dissolved NIA [26]. To further investigate this hypothesis, the depth profiles for PG in SC were also measured.

### 3.2. Depth Profiling of PG in the SC

**Figure 3:**
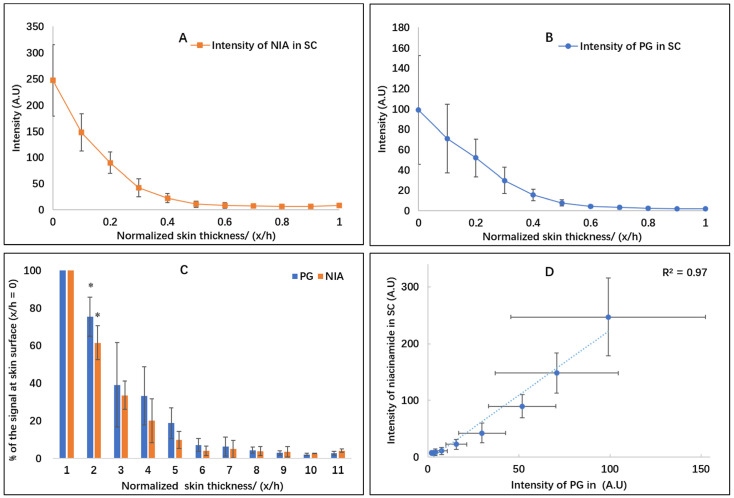
(**A**) Depth profile for the signal intensity of NIA in the SC; (**B**) depth profile for the signal intensity of PG in the SC; (**C**) normalized signal intensity percentage (%) of NIA and PG; and (**D**) correlation between the signal intensity of NIA and signal of PG in the SC with depth (mean ± SD, *n* = 6, * *p* < 0.05).

PG is one of the most widely used glycols in transdermal and topical formulations because of its favorable safety profile [35,41]. The signal intensity for the solvent PG in the SC was also analyzed using CRS, and the depth profile is shown in Figure 3. In line with the profiles of the NIA signal (Figure 3A), the profiles of PG were also exponential curves, reflecting the non-steady state diffusion of the solvent (Figure 3B). To facilitate the comparison of NIA and PG, their signal intensity was normalized as the recovered percentage of the respective signal at the skin surface (x/h = 0, Figure 3C). At x/h = 0.1, the signal of PG and NIA decreased to 75.3% and 61.5% of the signal at the skin surface, respectively. A significant reduction in intensity was detected for PG and NIA at x/h = 0.2, namely, 39.2% and 33.6%, respectively (*p* < 0.05). To examine the correlation between the distribution of vehicle and the active, the signals of NIA at various skin depths were plotted against the corresponding intensity values of PG. An excellent correlation (R^2^ = 0.97) was evident between the signal of the solvent and the active (Figure 3D). This suggests that the SC uptake of NIA appears to “track” the partitioning of PG into SC.

As noted, Pudney et al. [16] also used CRS to investigate in vivo percutaneous absorption of PG in humans. They measured the distribution of the model active *trans*-retinol in human skin as well as the solvent PG up to 10 h after application. Penetration of *trans*-retinol correlated well with the depth of penetration of PG, and this was consistent with later findings for monitoring of excipients in vivo with CRS by Mohammed et al. [17]. In this study, the distribution of the solvents PG and DMI as well as the active NIA was measured. An excellent agreement between the signal intensity of the solvents and the active in the skin was reported. Correlations between skin penetration of actives with PG where PG is the carrier solvent have been reported for a number of in vitro permeation studies [37,42]. Wotton et al. [42] investigated the percutaneous delivery of metronidazole in human skin using PG as a solvent. The cumulative permeation of both the permeant and solvent PG were determined. A correlation between metronidazole and PG permeation was reported. More recently, Haque et al. [37] reported in vitro permeation profiles of PG and the drug anthramycin. These studies also suggested that PG may be the carrier solvent for a range of actives during the process of percutaneous absorption.

### 3.3. Tape Stripping (TS) and Transepidermal Water Loss (TEWL)

**Figure 4:**
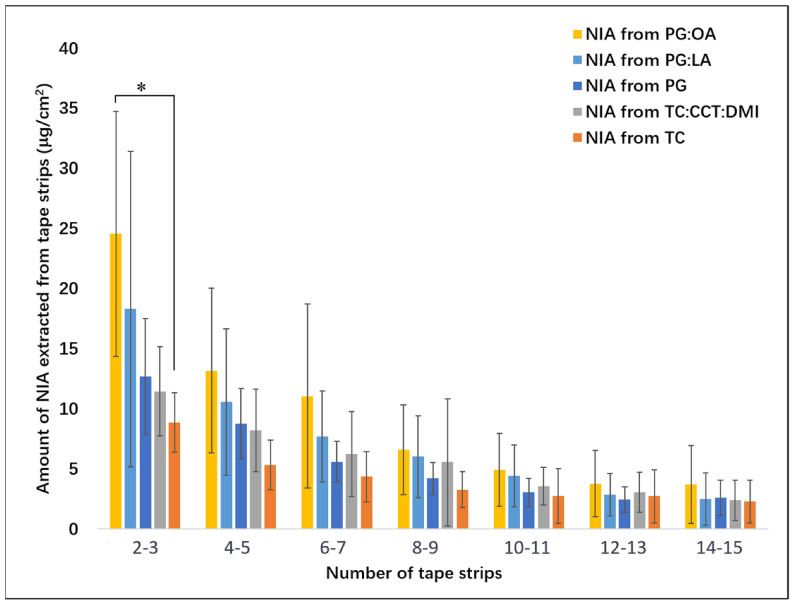
Amount of NIA (μg/cm^2^) removed from the volar forearm with depth following tape stripping (mean ± SD, *n* = 6, * *p* < 0.05).

Figure 4 summarizes the results from the TS studies. The amounts of NIA extracted for every two tapes are shown as a function of number of tapes stripped from the skin. For Tapes 2 and 3, significantly higher amounts of NIA were determined for PG:OA (24.6 ± 10.2 μg/cm^2^) compared with TC (8.9 ± 2.5 μg/cm^2^, *p* < 0.05). For PG, PG:LA and TC:CCT:DMI, the amounts of NIA extracted from Tapes 2 and 3 were determined as 18.3 ± 13.1, 12.7 ± 4.8 and 11.5 ± 3.7 μg/cm^2^, respectively, and no statistical differences were evident (*p* > 0.05). As for the total amount of NIA recovered from Tapes 2–15, a significantly higher value was noted for the binary PG:OA system compared with PG (*p* < 0.05, Table 1). The difference was consistent with the results of the in vivo CRS studies, where a higher AUC value for NIA was noted for the binary system in comparison with PG (Section 3.2). However, no statistical difference was evident when comparing PG with PG:LA (*p* > 0.05), which was not consistent with the CRS results reported above.

**Figure A1:**
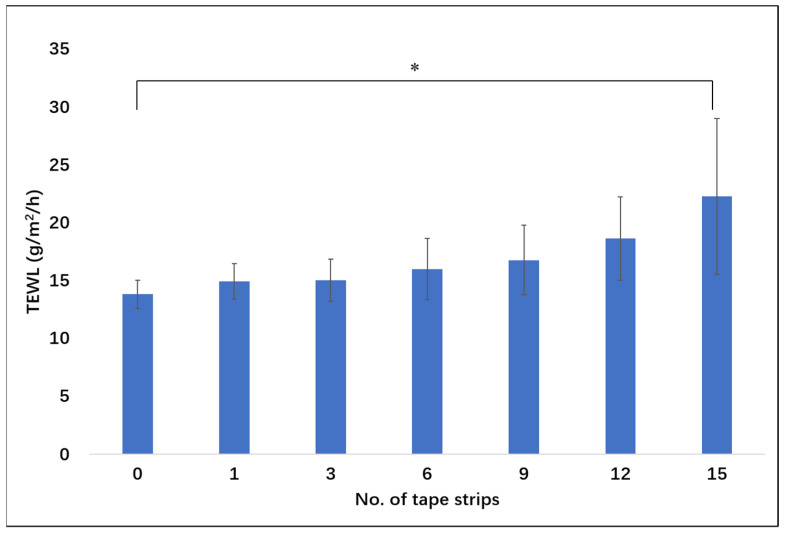
TEWL measurement of control site at mid volar forearm at baseline and after the removal of the first, third, sixth, ninth, twelfth and fifteenth tape (*n* = 6, mean ± SD). Statistical analysis is highlighted in the figure (* *p* < 0.05).

TEWL measurements were only performed at the control site to avoid any interference from applied solvents with the TEWL readings. As expected, TEWL values increased with increasing number of tape strips removed (Appendix A, Figure A1). Significant differences in TEWL values were noted for the baseline value compared with measurements following removal of Tape 15 (*p* < 0.05). No difference was evident when comparing the baseline values with readings taken for other tape strippings performed at the control sites (Figure A1, *p* > 0.05).

### 3.4. In Vitro–In Vivo Correlation

**Figure 5:**
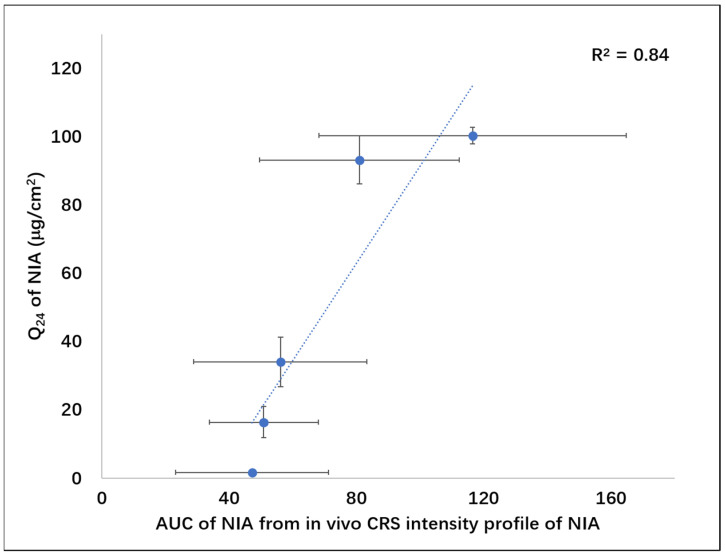
Area under the curve (AUC) for the depth profiles determined for CRS studies plotted against in vitro cumulative permeation of NIA at 24 h (Q_24_) in human skin (Pearson correlation coefficient, R^2^ = 0.84).

In previous in vitro permeation studies, the cumulative amounts of NIA that permeated from PG, TC, PG:OA, PG:LA and TC:CCT:DMI systems containing 5% NIA at 24 h were reported as 1.8 ± 0.4, 16.4 ± 4.6, 93.3 ± 7.1, 100.4 ± 2.5 and 34.1 ± 7.3 μg/cm^2^, respectively [25,26]. The amount of NIA that permeated at 24 h (Q_24_) was subsequently selected as an indicator of vehicle efficacy. The Q_24_ values were plotted against the corresponding AUC values from in vivo CRS intensity depth profiles of NIA reported in the present work (Figure 5). A good correlation (R^2^ = 0.84) was found between the AUC values for the in vivo CRS depth profiles and the in vitro data for NIA.

**Figure 6:**
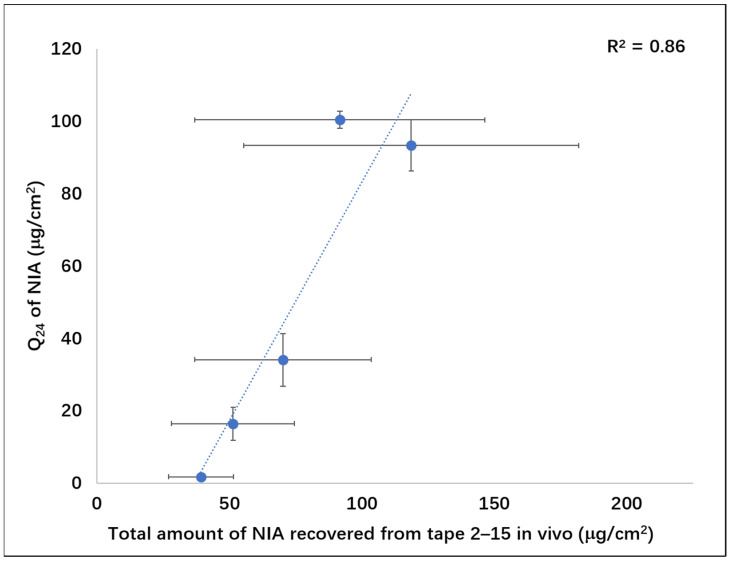
Total amounts of NIA determined from in vivo tape stripping studies (Tapes 2–15) and in vitro cumulative permeation of NIA at 24 h (Q_24_) in human skin (Pearson correlation coefficient, R^2^ = 0.86).

Additionally, a good correlation (R^2^ = 0.86) was evident between the in vitro cumulative permeation of NIA (Q_24_) and the total amount of NIA recovered from the in vivo TS studies (Figure 6). The correlation between data from in vitro Franz diffusion tests and in vivo tape stripping studies was also in line with findings reported by Ilić, et al. [43], who investigated the influence of “ready-to-use” formulation components (alkyl polyglucoside-mixed emulsifiers) on the microstructure of topical semisolid formulations, as well as critical formulation quality attributes, in vitro permeation and in vivo dermatopharmacokinetics of a model drug, aceclofenac (ACF). To assess whether in vitro and in vivo studies could differentiate the rate and extent of ACF skin delivery, Ilić and coworkers carried out Franz diffusion cell studies using porcine epidermis and in vivo tape stripping studies. The amounts of ACF that permeated through porcine epidermis at 30 h were plotted against the areas under ACF concentration depth profiles (AUC) from tape stripping after a 30 min application of the four tested formulations. A good in vitro–in vivo correlation (R^2^ = 0.98, *n* = 4) for skin permeation of ACF was reported.

Previously, Mohammed et al. [17] reported the results from an in vitro-in vivo correlation study of NIA using in vitro Franz diffusion cells and in vivo CRS studies [17]. The in vitro permeation studies were performed using human skin under infinite dose conditions for 24 h. The results were compared with the signal intensity of NIA measured at 4 μm in the volar forearm. A linear correlation between the signal intensity and the corresponding in vitro flux values of NIA was reported [17]. More recently, Iliopoulos et al. [30] published the first in vivo quantitative confocal Raman spectroscopy study. In vitro studies were performed under finite dose conditions for NIA solutions (5 μL/cm^2^) over 24 h. A dose of 5 μL/cm^2^ was also applied in the in vivo CRS study. An excellent correlation (R^2^ = 0.98) was found between the amounts of NIA measured per unit skin protein at a depth of 2 μm and the in vitro permeation of NIA from three formulations, namely TC, PG-propylene glycol monolaurate (PG-PGML) and PG-PGML-isopropyl myristate (PG-PGML-IPM). Additionally, to facilitate the comparison of CRS results published by different groups [20], Iliopoulos et al. also reported the skin uptake of NIA at 2 µm per unit area (cm^2^). A good correlation (R^2^ = 0.98) was also evident between the total amounts of NIA that permeated per unit area and the in vitro cumulative permeated amounts.

**Figure A2:**
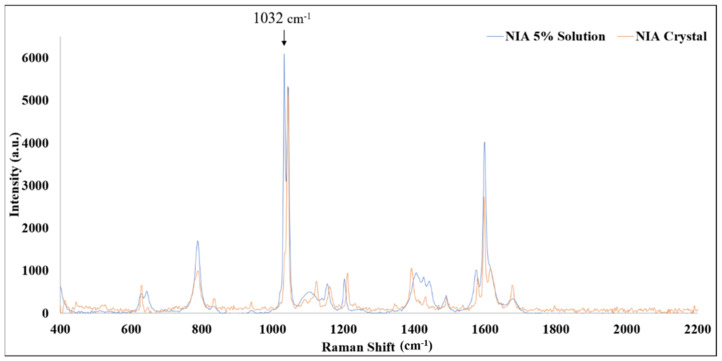
Raman spectra of crystalline NIA (red) and 5% NIA in water solution (blue). For NIA in solution, there were strong signals at Raman shift of 1032 cm^−1^ which were not noted for the Raman spectrum of crystalline NIA.

Although previous proof-of-concept CRS and quantitative CRS studies for NIA from our group have shown good correlations between in vitro and in vivo skin permeation, these studies were limited to only one subject for the in vivo studies [17,30]. Inter-subject variability of human skin is expected to complicate the correlation analysis [44,45]. The sample size in the present work was larger compared with previous in CRS experiments for NIA, increasing from one to six. The positive in vitro–in vivo correlation observed in the present work provides further evidence to support the results from our previous studies. Additionally, both in vivo CRS studies and TS experiments were performed in parallel, and the results obtained from the two techniques were comparable for all formulations. A good correlation for the skin permeation and TS data was also evident. However, as described above, TS is time consuming and laborious and contradictory TS results from different laboratories have been reported. In addition, there is debate concerning whether the first one or two tape strips should be discarded. The operating procedure of TS has to be standardized to ensure results obtained by this technique are reproducible. There are some other unresolved issues associated with TS. For example, furrows in the skin may complicate the interpretation of TS data. van der Molen, et al. [46] reported that the superficial layer of skin in the furrows remains non-stripped after removal of 20 tape strips. The residual compounds from the furrows may also affect the measurements of compound concentration profiles in the deeper layers [46]. The non-invasive nature of CRS coupled with rapid data collection underline this technique as a high-throughput and patient-friendly method for probing dermal delivery in vivo. Importantly, CRS is capable of distinguishing drug in solution versus crystalline NIA (Figure A2). This is likely to be a critical requirement for any analytical method to be taken forward for understanding the efficacy of topical formulations. As we previously noted, crystallized drug is not therapeutically available and remains “stranded” in the skin. In contrast, drug in solution is able to move through the SC and deeper layers [47]. To our knowledge, this is the first human study that investigated the effects of the combined use of PG and fatty acids on skin permeation. The positive in vivo results from PG:OA and PG:LA support a synergistic enhancement from combining PG with fatty acids for dermal delivery of actives.

## 4. Conclusions

In vivo permeation of the active NIA was investigated using both CRS and TS under finite dose conditions in human volunteers. For CRS studies, significantly higher signal intensity values of NIA were evident for the binary systems composed of PG and the fatty acids (OA and LA) compared with other formulations at a normalized skin depth of 0.3. These results were consistent with data obtained from previously published in vitro human skin permeation studies using Franz diffusion cells over 24 h. With reference to TS data, a higher value was noted for total penetrated amount of NIA from PG:OA compared with PG; no difference was evident comparing total NIA permeation from PG:LA and the other formulations. A very good correlation was noted between the skin distribution of the solvent PG and NIA from the CRS studies. Additionally, an excellent in vitro-in vivo correlation for AUC values from NIA Raman intensity profiles and NIA permeation in Franz cell diffusion studies was evident. In conclusion, the findings from the present work confirm that CRS is a powerful tool for the evaluation of drug and excipient dermatopharmacokinetics. Investigations of in vitro–in vivo correlations for other actives using the recently developed quantitative in vivo CRS method are ongoing. The ability of CRS to probe the viable epidermis is also expected to be advanced in the near future.

## Figures and Tables

**Table 1:** Total amounts of NIA extracted from Tapes 2–15 following tape stripping (mean ± SD, *n* = 6).

Solvent System	Amount of NIA (μg/cm^2^)
PG	39.3 ± 12.2
TC	51.4 ± 23.2
PG:OA (10:90)	118.6 ± 63.2
PG:LA (50:50)	91.6 ± 54.8
TC:CCT:DMI (50:25:25)	70.2 ± 33.2

## Data Availability

Data available on request.
